# Patient-specific, deliverable, and self-expandable surgical guide development and evaluation using 4D printing for laparoscopic partial nephrectomy

**DOI:** 10.1038/s41598-024-56075-5

**Published:** 2024-03-08

**Authors:** Junhyeok Ock, Eunseo Gwon, Taehun Kim, Sungchul On, Sojin Moon, Yoon Soo Kyung, Namkug Kim

**Affiliations:** 1 Department of Convergence Medicine, Asan Medical Institute of Convergence Science and Technology, Asan Medical Center, University of Ulsan College of Medicine, Seoul, Republic of Korea; 2 Department of Health Screening and Promotion Center, Asan Medical Center, University of Ulsan College of Medicine, 88 Olympic-ro 43-gil, Songpa-gu, Seoul, Korea; 3 Department of Radiology, Asan Medical Center, University of Ulsan College of Medicine, 88 Olympic-ro 43-gil, Songpa-gu, Seoul, Korea

**Keywords:** 3D printing, 4D printing, Minimally invasive, Laparoscopic partial nephrectomy, Surgical guide, Urological cancer, Cancer, Medical imaging

## Abstract

Accurate lesion diagnosis through computed tomography (CT) and advances in laparoscopic or robotic surgeries have increased partial nephrectomy survival rates. However, accurately marking the kidney resection area through the laparoscope is a prevalent challenge. Therefore, we fabricated and evaluated a 4D-printed kidney surgical guide (4DP-KSG) for laparoscopic partial nephrectomies based on CT images. The kidney phantom and 4DP-KSG were designed based on CT images from a renal cell carcinoma patient. 4DP-KSG were fabricated using shape-memory polymers. 4DP-KSG was compressed to a 10 mm thickness and restored to simulate laparoscopic port passage. The Bland–Altman evaluation assessed 4DP-KSG shape and marking accuracies before compression and after restoration with three operators. The kidney phantom’s shape accuracy was 0.436 ± 0.333 mm, and the 4DP-KSG’s shape accuracy was 0.818 ± 0.564 mm before compression and 0.389 ± 0.243 mm after restoration, with no significant differences. The 4DP-KSG marking accuracy was 0.952 ± 0.682 mm before compression and 0.793 ± 0.677 mm after restoration, with no statistical differences between operators (p = 0.899 and 0.992). In conclusion, our 4DP-KSG can be used for laparoscopic partial nephrectomies, providing precise and quantitative kidney tumor marking between operators before compression and after restoration.

## Introduction

Despite escalated kidney tumor prevalence, mortality rates have declined in developed countries due to imaging technology advancements and early screening advantages^1^. As chemotherapy is often ineffective, kidney tumor surgery is preferred through excision^2^; therefore, various surgical methods are continuously being developed. Since laparoscopic or robot surgeries were introduced in the twentieth century, minimally invasive surgeries have significantly progressed^3,4^. Furthermore, partial nephrectomy is a substantial proportion of kidney cancer treatment, and patient survival rates have elevated from laparoscopic or robotic surgeries^5,6^. In addition, kidney tumors can be accurately diagnosed using medical imaging devices such as CT and MR, increasing surgery efficacy and patient management^7,8^. However, methods for accurately marking the tumor area determined through medical imaging inside the operation room are currently lacking. Furthermore, kidney tumors must be meticulously excised within 20–30 min, as tumors are only removed after blocking the kidney’s arterial and venous supply. This blood circulation deprivation can readily eradicate kidney cells without swift recirculation^9^.

3D printing (3DP) technology is broadly implemented across various fields because it effortlessly fabricates complex shapes. For example, its medical field applications include educational simulators for patients and trainees, surgical guides, and implants^10^. Furthermore, many studies targeting assorted cancer types, including breast, skin, and bone, utilize surgical guides as they can mark the resection plan obtained through medical imaging directly onto the patient^11–13^. In addition, several studies have achieved accurate partial nephrectomies by developing surgical plans using 3DP renal cancer modeling based on MRI data before laparoscopic surgery^14,15^. However, these studies cannot directly mark the accurately established surgical plan onto the patient during the operation, making these methods impractical for immediate laparoscopic surgery.

Similarly, 4D printing (4DP) is used in medical fields for its shape-memory polymers (SMPs). For instance, a 4DP pediatric tracheobronchomalacia relief device was designed to prevent airway obstruction through structural variations using biocompatible and absorbable materials that do not interfere with growth^16^. Additionally, 4DP scaffolds for tissue engineering can seed cells at temporary shape scaffolds and restore them to a permanent form by placing them in real tissue^17^. Self-fitting bone implants are molded using mixed polylactide (PLA)/hydroxyapatite (HA), which can maintain a temporary shape for three compression-heating-compression cycles, and have achieved a 98% shape recovery rate at 70 °C^18^.

Therefore, we developed and evaluated a patient-specific, transportable, and self-expandable surgical guide using 4D printing with SMP materials based on medical imaging for laparoscopic partial nephrectomies.

## Materials and methods

### Overall process

**Figure 1:**
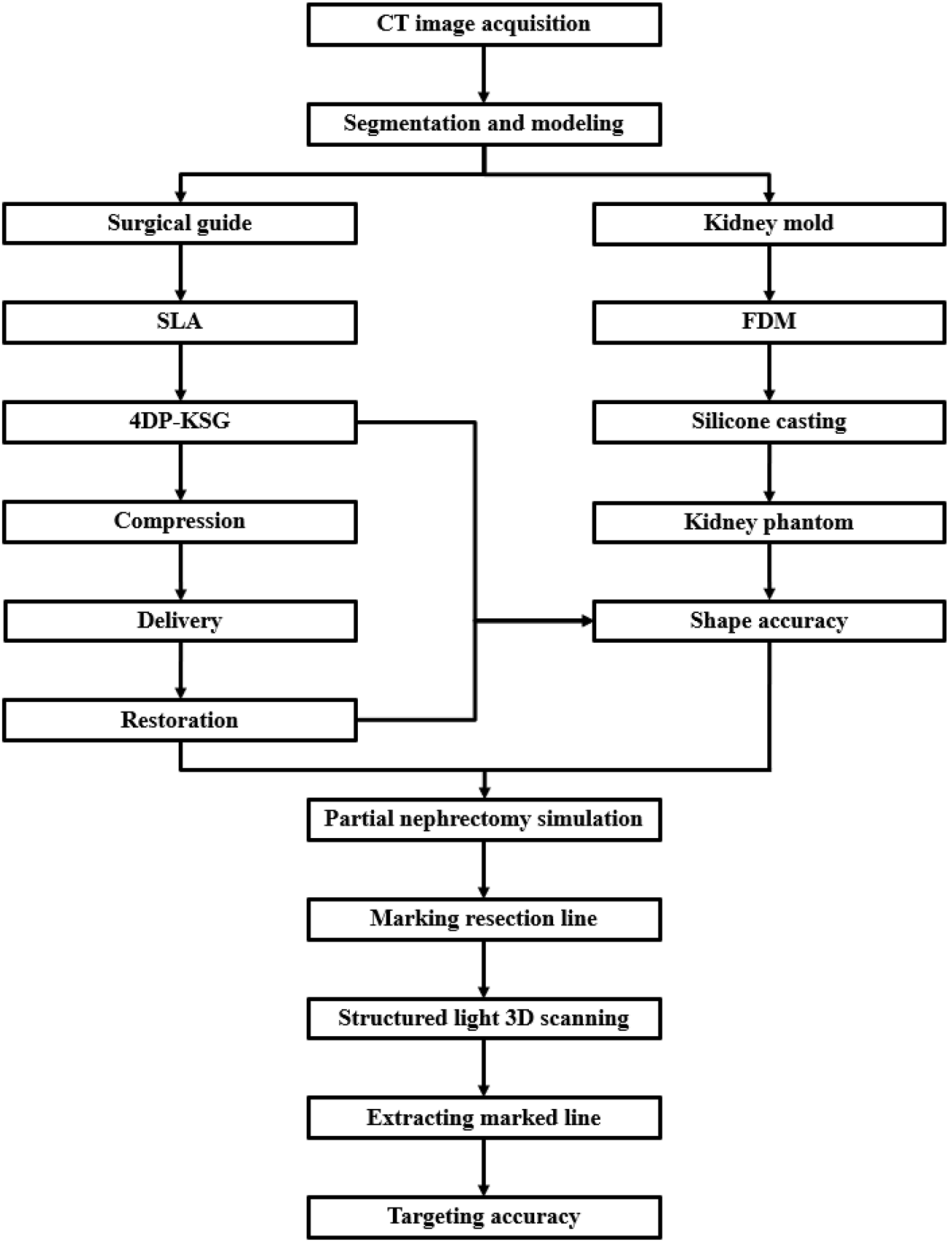
4D-printed kidney cancer resection guide fabrication and evaluation. *CT* computed tomography, *SLA* stereolithography apparatus, *FDM* fused deposition modeling, *4DP-KSG* 4D-printed kidney surgical guide.

Figure 1 conveys the overall patient-specific 4DP-KSG development and evaluation. Based on a patient's medical images, the 4DP-KSG and kidney phantom were designed and fabricated using 3DP technology with post-processing and silicone casting. The 4DP-KSG incorporated 4D printing technology, and compression, delivery, and restoration processes were required to mark the tumor resection line. The 4DP-KSG was evaluated before compression and after restoration to determine whether the shape had changed. Finally, we assembled the 4DP-KSG onto the kidney phantom to verify its targeting accuracy.

### Image acquisition and pre-processing

**Figure 2:**
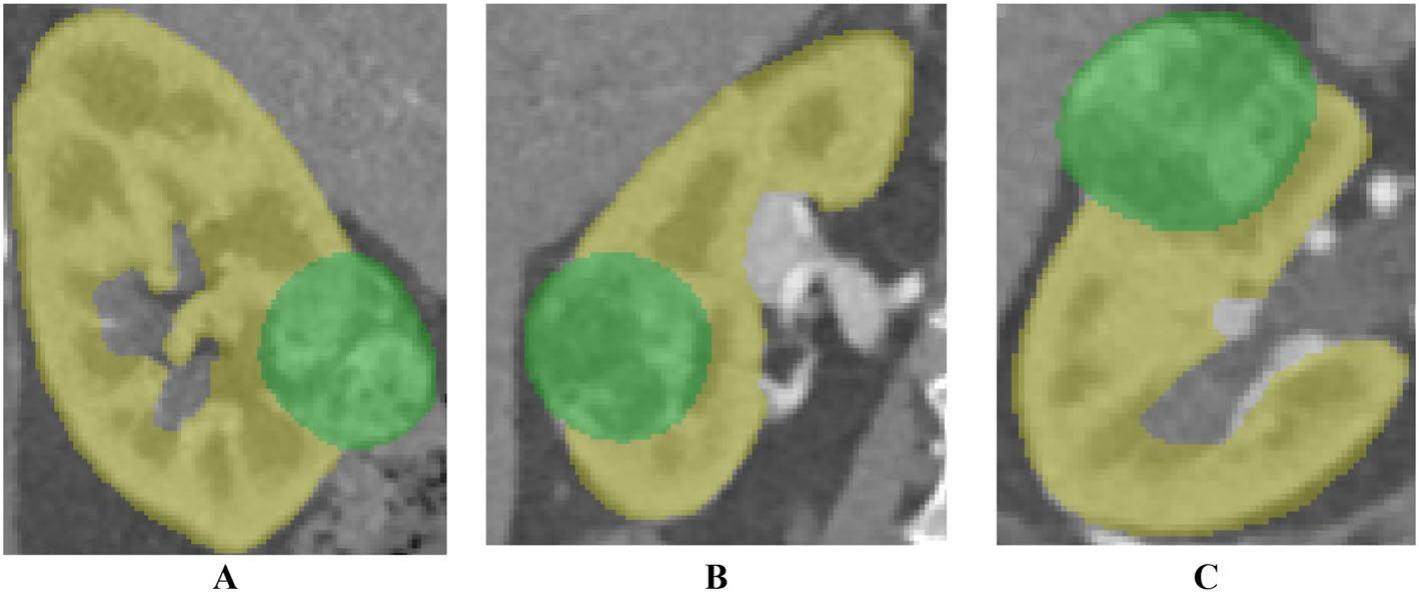
Segmented parenchyma and tumor (**A**) sagittal, (**B**) coronal, and (**C**) axial views from multi-detector computed tomography images of a 61-year-old female patient diagnosed with renal cell carcinoma (RCC).

This study received approval from the Institutional Review Board of the Asan Medical Center (IRB No. 2021-0449) and adhered to the Declaration of Helsinki principles. Anonymized images scanned with multi-detector computed tomography (SOMATOM Definition Edge, Siemens Healthcare, Erlangen, Germany) using a 120 kVp tube voltage and a 1mm slice thickness and written informed consent were obtained from a 61-year-old female patient diagnosed with renal cell carcinoma (RCC). Tumor and parenchyma segmentations were implemented using Mimics ver. 17 (Materialise Inc., Leuven, Belgium) with − 126 to 360 and − 196 to 407 Hounsfield units (HU) thresholding, followed by an expert’s manual modifications (Fig. 2).

### Phantom and guide models

**Figure 3:**
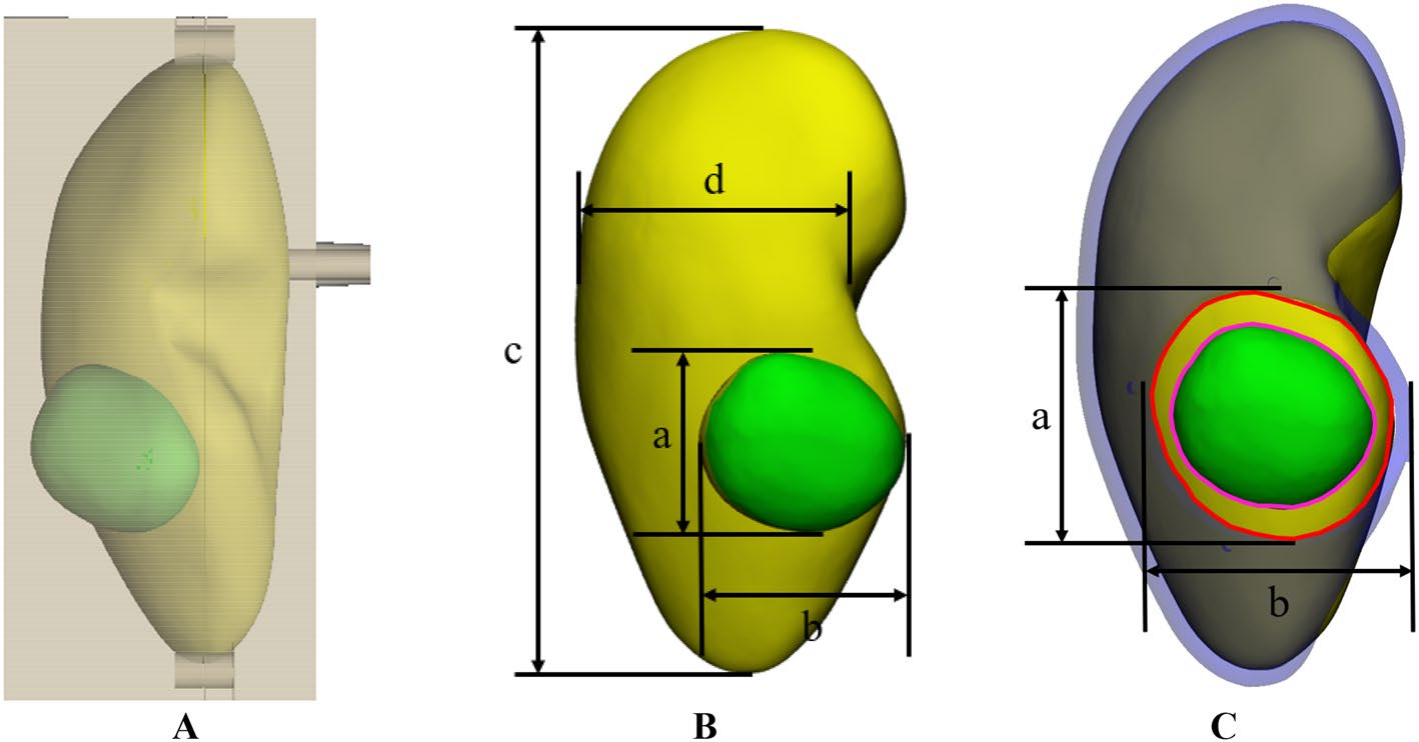
The designed kidney phantom mold and 4D-printed kidney surgical guide (4DP-KSG). (**A**) The assembled kidney molds. (**B**) The kidney phantom model and measurements for fabrication accuracy evaluation: (a) tumor height, (b) tumor width, (c) parenchyma height, and (d) parenchyma width. (**C**) The assembled 4DP-KSG on the kidney phantom with outlined tumor (pink), safety margin (red), and measurements for shape accuracy evaluation: (e) resection line height, (f) resection line width, (g) 4DP-KSG height, and (h) 4DP-KSG width.

Based on the patient's CT images, the patient-specific 4DP-KSG and phantom were modeled using 3-Matics ver. 9 (Materialise Inc., Leuven, Belgium). Only the right kidney retained the tumor and was included in this study. The kidney mold was designed as a negative parenchyma and tumor-shaped body model, then divided into top and bottom sections. A release agent was applied to the mold, and the top and bottom molds were assembled. Next, silicone was cast into the kidney mold and left to cure for one day. Finally, the kidney phantom was obtained by removing the mold. The 4DP-KSG was designed based on the segmented tumor, parenchyma, and surgeon-recommended 5 mm safety margin. The 4DP-KSG was modeled to encompass the entire region, including both parenchyma extremes, and a medical dermal marking pen designated the incision points and safety resection margin (Fig. 3).

### Phantom and 4DP-KSG fabrication

**Figure 4:**
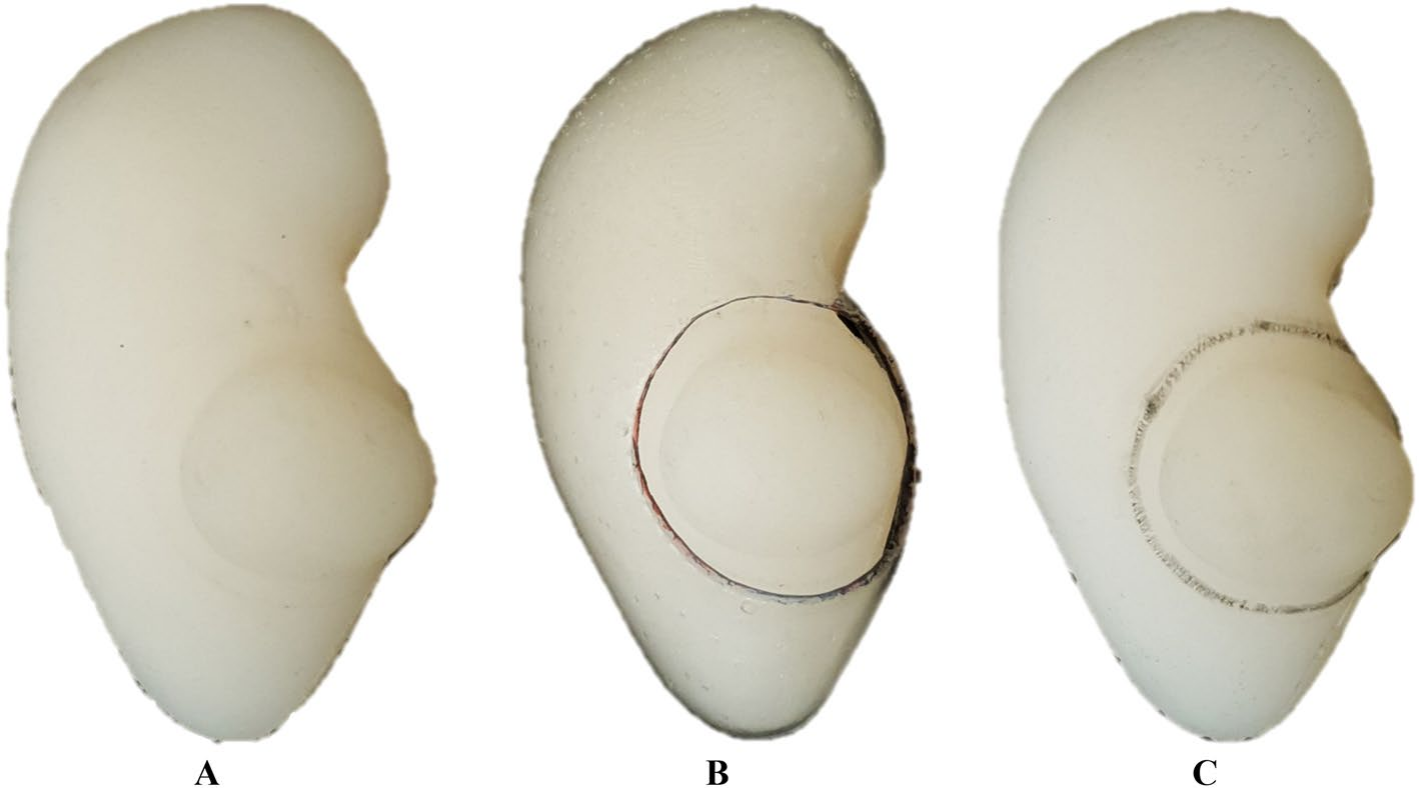
The fabricated kidney phantom with a 4D-printed kidney surgical guide (4DP-KSG). (**A**) Fabricated kidney phantom. (**B**) Fabricated kidney phantom assembled with 4DP-KSG. (**C**) Marked resection line on the kidney phantom using 4DP-KSG.

Each mold was created using fused deposition modeling with polylactic acid filament (DP200, Sindoh, Korea) to ensure it did not break when the silicon was cast. In addition, we used ecoflex00-30 silicone for the kidney phantom because its 170 kPa elastic modulus was closest to the human kidney’s 180.32 kPa, indicating similar mechanical properties^19,20^ (Fig. 4A). Finally, the 4DP-KSG was fabricated using a stereolithography apparatus (SLA) (A1, Sindoh, Seoul, Korea), SMP resin (TC-85DAC, Graphy, Seoul, Korea), and a curing machine for 15-min post-curation (U102H, Graphy, Seoul, Korea) (Fig. 4B). Kidney phantoms and 4DP-KSG were fabricated each only single to maintain consistency.

### 4D printing and shape programming

**Figure 5:**
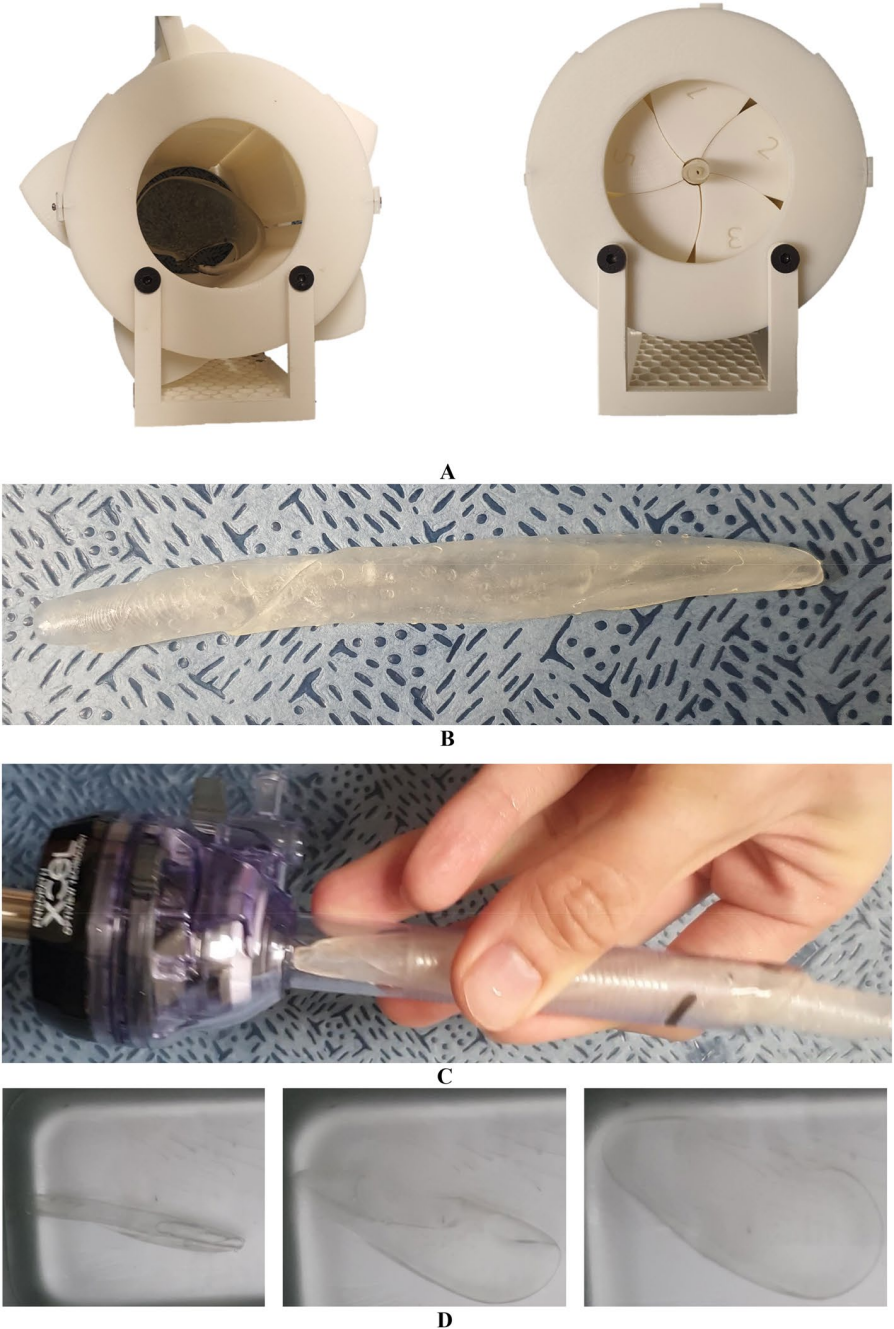
Overall programming procedure for the 4D-printed kidney surgical guide (4DP-KSG). (**A**) 4DP-KSG compression. (**B**) Compressed 4DP-KSG. (**C**) 4D-KSG passed through the laparoscopic port. (**D**) 4DP-KSG restoration.

4DP-KSG’s fabrication and the laparoscopic nephrectomy application were as follows:

1. The 4D-KSG was fabricated using SLA with SMP resin, guaranteeing a permanent shape to guide the nephrectomy resection line.
2. The 4DP-KSG was soaked in a 70 °C water bath and compressed to less than a 10 mm thickness and 10 mm inner diameter using a separately fabricated compressor with a shape similar to a camera iris aperture (Fig. 5A).
3. Subsequently, the guide was cooled in a 10 °C water bath to retain its temporary shape (Fig. 5B) and passed through the laparoscopic port (ENDOPATH Xcel^®^ Optiview^®^ trocar, Ethicon, Somerville, NJ, USA) (Fig. 5C).
4. The 4DP-KSG was restored to its original permanent shape by soaking the guide in a 70 °C water bath (Fig. 5D). All procedures were recorded (Supplemental Video 1).

### Evaluations and statistical analysis

The kidney phantom’s shape accuracy was prioritized before measuring 4DP-KSG’s marking accuracy; thus, we first measured the phantom’s four designated landmarks (Fig. 3B). Three operators took height and width measurements from the standard tessellation language (STL) and fabricated models’ parenchyma and tumor five times using the 3-Matic and vernier calipers, respectively (Fig. 3B). However, a tumor resection line can only be provided after the 4DP-KSG shape programming process. Therefore, we compared 4DP-KSG’s shape accuracy before compression and after restoration.

Next, three operators measured the height and width of the 4DP-KSG and resection lines five times using 3-Matic software and vernier calipers (Fig. 3C). 4DP-KSG’s marking accuracy was evaluated before compression and after restoration, as an accurate resection line is vital. Three operators also marked the resection line on the kidney phantom three times using the 4DP-KSG and a medical dermal marking pen (Fig. 4C), scanning the marked kidney phantom with a structured light 3D scanner (EinScan Pro 2X 2020, SHINING 3D Tech.Co., Ltd., Hangzhou, China). The scanned kidney phantom was converted into an STL file, and all files were registered with the global registration function, including manual corrections based on the modeled phantom CT image. Finally, resection lines were extracted from the scanned models for comparison. We extracted 31 corresponding points from each line and compared them using the Harsdorf distance^21^. 4DP-KSG’s marking accuracy measuring was conducted on one simulator and 4DP-KSG in a reuse manner by erasing the resection line drawn on the kidney phantom using an oil-based solution after the digitization of the resection line and kidney phantom was completed.

A Bland–Altman analysis using Med-Calc ver. 19 (MedCalc Software Ltd., Acacialaan, Belgium) evaluated the kidney phantom’s and 4DP-KSG’s shape and marking accuracies before compression and after restoration. In addition, the Mann–Whitney *U* test also evaluated 4DP-KSG’s marking and shape accuracies using IBM SPSS Statistics v25.00 (IBM Corp., New York, USA). Lastly, the one-way analysis of variance (ANOVA) compared operator differences utilizing IBM SPSS Statistics v25.00.

## Results

### Kidney phantom shape accuracy

**Figure 6:**
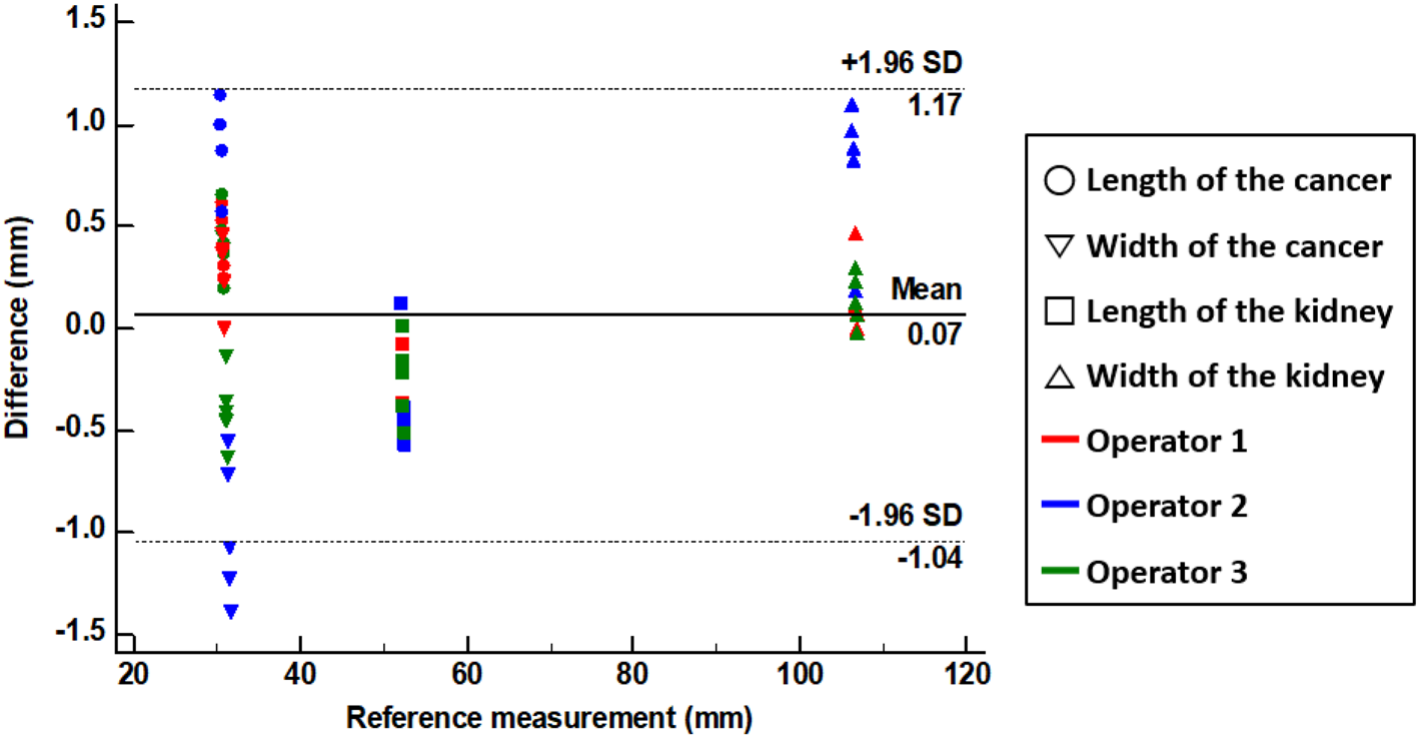
Bland–Altman plot to evaluate measurement errors between the standard tessellation language (STL) model and the fabricated kidney phantom. *SD* standard deviation.

Figure 3B illustrates our error measurements (mean ± SD) for tumor height (0.535 ± 0.284 mm), tumor width (0.559 ± 0.394 mm), parenchyma height (0.368 ± 0.383 mm), and parenchyma width (0.284 ± 0.169 mm), and the kidney phantom’s overall measurement error was 0.436 ± 0.333 mm (limit of agreement from − 1.09 to 1.22 mm). All measurements, except for a few tumor widths, were within the 95% limit of agreement (Fig. 6).

### 4DP-KSG shape accuracy

**Figure 7:**
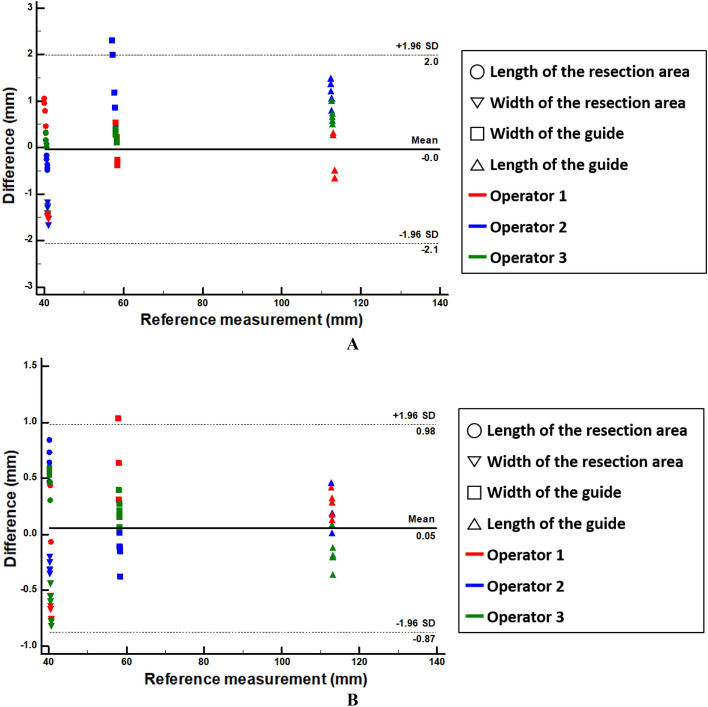
Bland–Altman plot to evaluate differences between the standard tessellation language (STL) model and the 4D-printed kidney surgical guide (**A**) before compression and (**B**) after restoration.

We used the Bland–Altman plot to evaluate measurement errors between the STL model and the fabricated 4DP-KSG guide before compression and after restoration. The 4DP-KSG measurement error was − 0.03 ± 0.99 mm before compression (limit of agreement from − 2.19 to 2.11 mm) and 0.05 ± 0.45 mm after restoration (limit of agreement from − 0.85 to 0.95 mm) (Supplemental Table 1). All measurements, except for a few guide widths, were within the 95% limit of agreement (Fig. 7A,B). There was no statistical difference in 4DP-KSG’s shape accuracy before compression and after restoration (p = 0.479).

### 4DP-KSG marking accuracy

**Figure 8:**
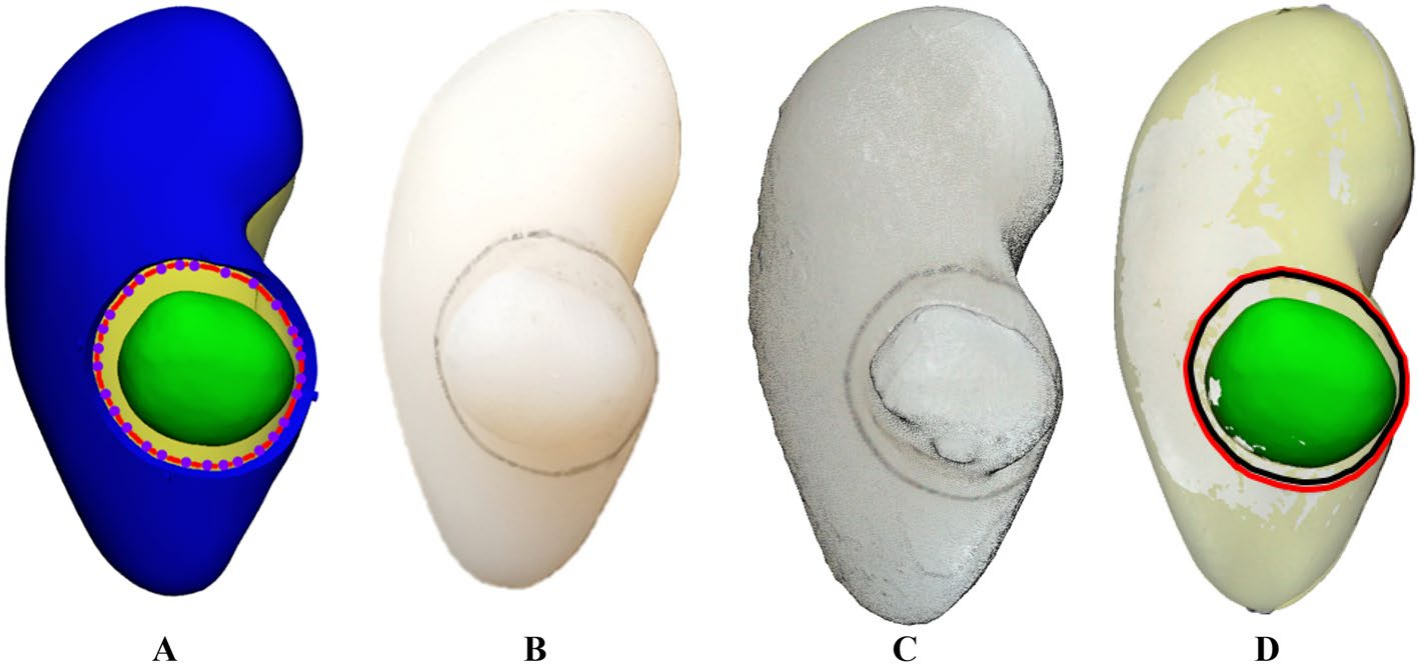
Planned and marked resection lines using the 4D-printed kidney surgical guide (4DP-KSG). (**A**) The planned resection line (red) and 31 corresponding points (purple dot) on the kidney phantom assembled with 4DP-KSG, (**B**) the kidney phantom’s marked resection line, (**C**) the scanned kidney phantom’s marked resection line, and (**D**) the model’s matching planned resection (red) and marked resection lines (black).

**Figure 9:**
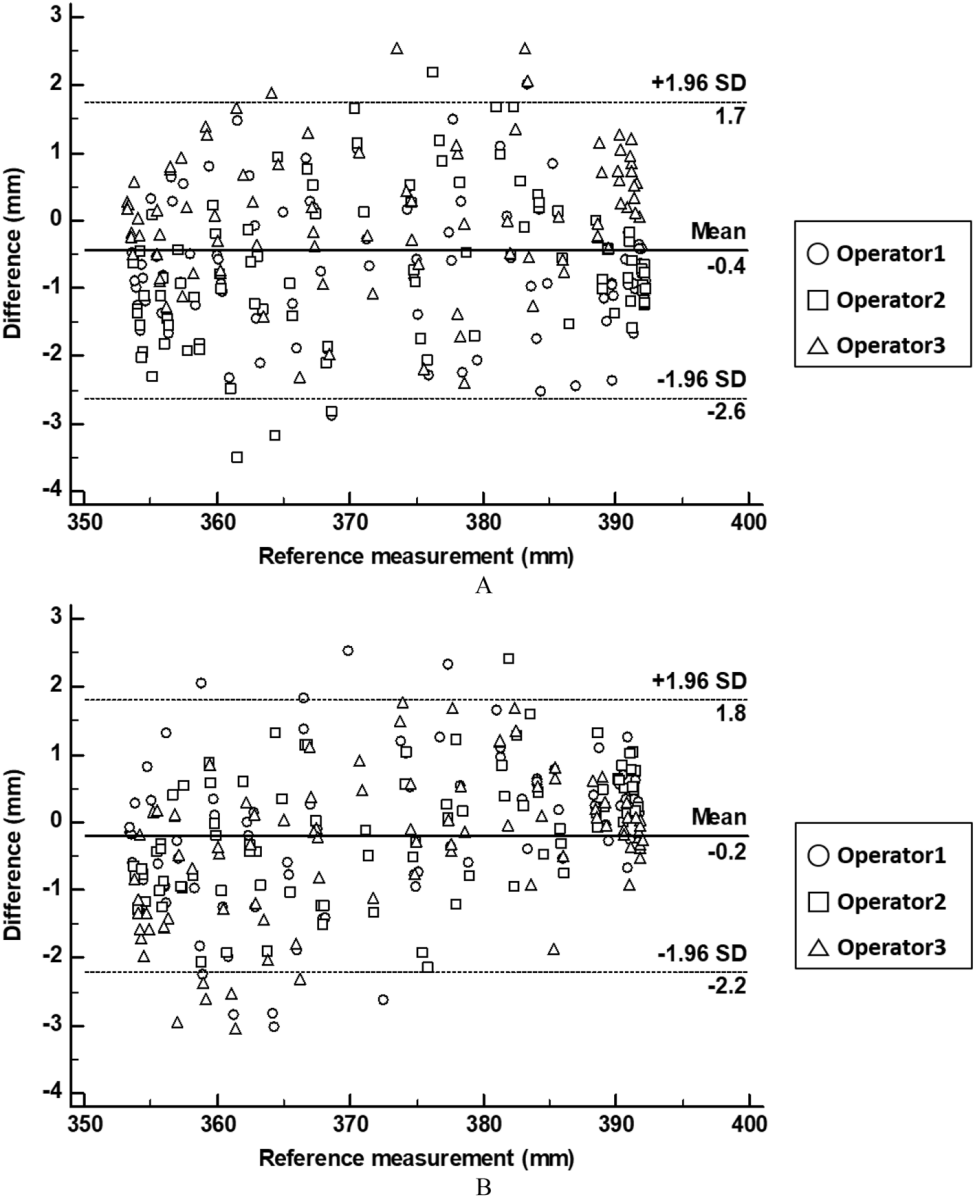
Bland–Altman plot to evaluate marking accuracy differences (**A**) before compression and (**B**) after restoration. *SD* standard deviation.

The marked and planned resection lines were extracted and compared for marking accuracy evaluation. Measurements from before compression, after restoration, three operators, and three repetitions resulted in 18 resection lines. Next, each line comprised 31 corresponding points measuring its distance from the origin point; thus, the Bland–Altman plot evaluated 558 points between planned and marked lines (Fig. 8). The marking accuracy measurement errors were 0.952 ± 0.682 mm before compression (limit of agreement from − 2.60 to 1.70 mm) and 0.793 ± 0.677 mm after restoration (limit of agreement from − 2.20 to 1.80 mm) (Fig. 9). There was no statistical difference among operators between resection lines before compression and after restoration (p = 0.899 and 0.992).

## Discussion

Laparoscopic or robotic surgeries have increased and continue to increase partial nephrectomy survival rates through scientific and surgical skill advancements^3,4,6,9,22^. However, nephrectomies require clamping of the renal artery and vein, so the operation must be completed within 20–30 min^9,23^. Although CT and MR medical imaging allow accurate diagnoses, methods for directly explaining surgical procedures to patients within operating rooms are lacking^7,8^. Thus, partial nephrectomies require minimally invasive, rapid, and accurate lesion removal using laparoscopic or robotic surgery. Therefore, we developed and evaluated a patient-specific, deliverable, and self-expandable surgical guide for laparoscopic partial nephrectomies using 4DP.

The 4DP-KSG reduces surgery duration while increasing accuracy by marking the resection line directly onto the patient's kidney before clamping blood vessels. Furthermore, the programmed 4DP-KSG can pass through the laparoscopic port and be restored to its original shape with heated water, indicating potential laparoscopic or robotic surgery applications. The 4DP-KSG used for laparoscopic or robotic surgery must be able to pass the trocar and restore it to its predefined shape. These characteristics are critical for the guide's clinical applicability, effectiveness, and outcome predictability. Therefore, technical applicability was verified by measuring and evaluating the overall shape and tumor-targeting accuracy of the guide before compression and after restoration. We fabricated a realistic kidney phantom using ecoflex00-30 silicone, which expresses mechanical properties similar to a human kidney, to evaluate 4DP-KSG’s marking accuracy^19,20^. The phantom’s shape accuracy was within reasonable error (limit of agreement from − 1.09 to 1.22 mm). Similarly, 4DP-KSG’s shape accuracies before compression and after restoration were also within reasonable error (limits of agreement ranging from − 2.20 to 2.10 mm and − 0.94 to 1.04 mm), exhibiting no statistical differences between guides. In addition, measurement errors in marking accuracy before compression and after restoration were within reasonable bounds (limit of agreement from − 2.60 to 1.70 mm and − 2.20 to 1.80 mm) and had no statistical differences among operators (p = 0.899 and 0.992).

These results substantiate that novice surgeons can obtain accurate and identical surgical results using 4DP-KGS. Comparatively, dental implant placement surgical guides anchored to teeth and gums report an approximate 1–2 mm accuracy^24,25^. In addition, surgical guides fixed to soft tissues, such as breasts and skin, have indicated a 2 mm accuracy^12,13^. These results verify that 4DP-KSG could be used for clinical applications.

Our study has several limitations. First, this study did not evaluate marking accuracy for various tumor types, locations, and sizes. However, follow-up studies will incorporate more realistic kidney phantoms representing these variations. Second, unlike a natural surgical environment, our marking accuracy was measured with a wide field of view. An environment with a constrained field of view can reduce accuracy; thus, subsequent studies will consider these environmental conditions. Third, the resection line was not marked using laparoscopy. Unlike hand movement, laparoscopy limits movement and often reduces marking accuracy. In a follow-up study, marking accuracy will be verified using laparoscopy. Fourthly, 4D-KSG is activated at 70 °C, a temperature likely challenging to use within the human body. Therefore, we will also research materials and guide structures easily deformed at lower temperatures. Fifth, the efficacy of the 4DP-KSG has not yet been compared with existing tumor targeting methods, such as intraoperative ultrasonography. In a follow-up study, we aim to conduct a comparison, focusing on the accuracy and clinical utility of our surgical guides comparing to other guiding methods including ultrasound technology, robot-assisted partial nephrectomy, and 3D-printed kidney cancer models^14,26^. Sixth, our study was not evaluated to actual clinical application. We aimed to evaluate the usefulness of the guide before applying 4DP-KSG to patients as a pre-clinical study. In a follow-up study, we will plan to apply 4DP-KSG for a patient. 4DP-KSG based laparoscopic or robotic nephrectomy was as follows: First, sterilized 4DP-KSG using ethylene oxide gas was inserted into the patient's body through the laparoscopic port and intragastric balloon. Second, by inserting 40 °C water into the intragastric balloon, 4DP-KSG restored its original shape. Last, the restored 4DP-KSG is attached to the kidney to mark the surgical plan directly on the patient's body, and then it is removed after being cut into pieces. Lastly, only medical 3D printing experts participated in these experiments. Future in-vivo studies will include various surgeon levels.

## Conclusion

Our patient-specific, deliverable, and self-expandable 4DP-KSG demonstrated precise and quantitative kidney tumor targeting with no significant variability between operators, compression, and restoration. Therefore, our method effectuates an accurate, effective, and minimally invasive tumor removal during partial nephrectomy.

### Supplementary Information

Supplementary Table 1.

Supplementary Video 1.

## Data Availability

The datasets generated in this study during the current study are not publicly available because the data used in our study were created based on patient images but are available from the corresponding author on a reasonable request.

## Abbreviations

CT: Computed tomography
4DP-KSG: 4D-printed kidney surgical guide
3DP: 3D printing
4DP: 4D printing
SMPs: Shape-memory polymers
PLA: Polylactide
HA: Hydroxyapatite
SLA: Stereolithography apparatus
FDM: Fused deposition modeling
RCC: Renal cell carcinoma
HU: Hounsfield units
STL: Standard tessellation language
ANOVA: Analysis of variance
