# Supplementary material for: Patient-specific, deliverable, and self-expandable surgical guide development and evaluation using 4D printing for laparoscopic partial nephrectomy
Source: Sci Rep. 2024 Mar 8;14:5722. doi: 10.1038/s41598-024-56075-5 (PMC10924080; doi:10.1038/s41598-024-56075-5)
Supplement: Supplementary file 1 — Supplementary Table 1. [file 41598_2024_56075_MOESM1_ESM.pdf]

Measurements	Before compression		After restoration		p*
	Mean	SD	Mean	SD	
**Resection line height**	**−1.44**	**−1.70 ~ −1.18**	**−0.55**	**−0.94 ~ −0.15**	**0.07**
**Resection line width**	**0.17**	**−0.79 ~ 1.12**	**0.50**	**0.11 ~ 0.91**	**0.13**
**4DP-KSG height**	**0.56**	**0.56 ~ 1.88**	**0.09**	**−0.36 ~ 0.56**	**< 0.01**
**4DP-KSG width**	**0.56**	**−0.91 ~ 2.03**	**0.15**	**−0.53 ~ 0.84**	**0.07**
**Overall shape**	**−0.03**	**−2.19 ~ 2.11**	**0.05**	**−0.85 ~ 0.95**	**0.19**

Supplemental Table 1. The 4D-printed kidney surgical guide's shape accuracy before compression and after restoration. (SD, standard deviation; *, Mann–Whitney U test)
